# Impact of COVID-19 pandemic on the dynamic of patients with oral and maxillofacial trauma: interrupted time-series analysis

**DOI:** 10.1038/s41598-024-63890-3

**Published:** 2024-06-08

**Authors:** Hiroto Tatsumi, Yuhei Matsuda, Tatsuo Okui, Masaaki Karino, Takashi Koike, Satoe Okuma, Erina Toda, Shinji Ishizuka, Rie Sonoyama-Osako, Reon Morioka, Tatsuhito Kotani, Yukiho Shimamura, Takahiro Kanno

**Affiliations:** 1 Department of Oral and Maxillofacial Surgery, Shimane University Faculty of Medicine, 89-1 Enya-cho, Izumo, Shimane 693-8501 Japan; 2 Maxillofacial Trauma Center, Shimane University Hospital, Izumo, Shimane Japan; 3 Department of Oral and Maxillofacial Surgery, Shimane Prefectural Central Hospital, Izumo, Shimane Japan; 4 Department of Oral and Maxillofacial Surgery, Unnan City Hospital, Unnan, Shimane Japan; 5 Department of Oral and Maxillofacial Surgery, National Hospital Organization Hamada Medical Center, Hamada, Shimane Japan; 6 Division of Oral and Maxillofacial Surgery, Oki Hospital, Oki, Shimane Japan; 7 Department of Oral and Maxillofacial Surgery, Masuda Red Cross Hospital, Masuda, Shimane Japan

**Keywords:** Epidemiology, Dental diseases, Oral diseases

## Abstract

Oral and maxillofacial trauma is influenced by various factors, including regional characteristics and social background. Due to the coronavirus disease 2019 (COVID-19) pandemic, a state of emergency was declared in Japan in March 2020. In this study, we aimed to examine the dynamics of patients with oral and maxillofacial trauma over a 12-years period using interrupted time-series (ITS) analysis. Patients were examined at the Shimane University Hospital, Maxillofacial Trauma Center from April 2012 to April 2023. In addition to general patient characteristics, data regarding the type of trauma and its treatment were obtained from 1203 patients (770 men and 433 women). Group comparisons showed significant differences in age, trauma status, method of treatment, referral source, route, and injury occasion. ITS analysis indicated significant changes in combined nasal fractures, non-invasive reduction, and sports injuries (*P* < 0.05), suggesting COVID-19 significantly impacted oral and maxillofacial trauma dynamics. A pandemic of an infectious disease may decrease the number of minor trauma cases but increase the number of injuries from outdoor activities, resulting in no overall change in the dynamics of the number of trauma patients. Medical systems for oral and maxillofacial trauma should be in place at all times, independent of infectious disease pandemics.

## Introduction

The novel coronavirus, severe acute respiratory syndrome coronavirus 2, was first discovered in China in 2019 and causes coronavirus disease 2019 (COVID-19)^1^. Due to its high infectivity and global spread, a pandemic was declared by the World Health Organization in 2020^2^. Preventive measures included mask wearing, hand washing, social distancing, and implementation of policies to restrict the behavior of citizens. Many reports have indicated that lockdowns and emergency declarations are crucial on the dynamics of disease outbreaks and the health care system and drastically alter people’s daily lives^3,4^.

Behavioral restrictions during the COVID-19 pandemic disproportionately affected socially vulnerable populations, leading to increased depressive symptoms and social frailty among the elderly due to stay-at-home orders^5^. A retrospective study in internal medicine of 748 Japanese patients with chronic diseases revealed changes in office and at-home blood pressures after the state of emergency declaration. Among these patients, 58% expressed concerns about the negative effects of hypertension, while 39% and 17% reported decreased physical activity and deteriorating diet, respectively^6^. Neurosurgical departments experienced a decline in outpatient visits and surgeries, potentially impacting teaching cases and treatment strategies, especially in subspecialized areas^7^. An investigation of pediatric dentistry showed no change in the sex distribution post-COVID-19, but the age distribution increased for patients under 6 years old. Significant changes occurred in diagnostic names, such as caries, retained primary teeth, malocclusion, and deep periorbital fissures, whereas studies on pulpitis, apical periodontitis, tooth trauma, early loss of primary teeth, and excess teeth reported minimal changes^8^.

Oral and maxillofacial trauma includes superficial lacerations, abrasions, and maxillofacial bone fractures. Fractures in oral and maxillofacial trauma occur primarily in young people and are more prevalent in males. Traffic accidents, sports, violence, and falls are common causes of fractures in oral and maxillofacial trauma^9^. Sports-related traumatic fractures are common among adolescents and young adults^10^. The location of fractures varies by age, with maxillofacial bone fractures being more common in young adults, whereas fractures of the jaw and facial bones are more common in older adults^11^. Beyond aesthetic implications, oral and maxillofacial trauma can affect physical function, impacting trigeminal and facial nerves. Early treatment is crucial for preserving the patient’s quality of life^12^.

Several reports on the impact of the COVID-19 pandemic in oral and maxillofacial trauma have been published^12–16^. A report from the United States indicated that during the first 7 weeks of the pandemic lockdown, the number of maxillofacial trauma cases and interpersonal violence-related cases decreased^13^. Another report from the United States found a decrease in oral–maxillofacial trauma cases during the pandemic; however, the maxillofacial and systemic injuries were more severe^14^. A report from the United Kingdom highlighted changes in oral and maxillofacial injury patterns during the lockdown, with altered dynamics and deferred treatment of oral and maxillofacial fracture cases, and 14% of patients being treated differently than in usual practice^15^. In Germany, the number of trauma cases increased in private clinics, with an increase in alcohol-related trauma cases^16^. A multicenter study reported that the common striking similarities were increased among patients with facial trauma from falls and decreased among cases of interpersonal violence-related facial trauma, indicating that COVID-19-related public health measures altered the epidemiological characteristics of oral and maxillofacial trauma^17^.

However, a literature review on the impact of the COVID-19 pandemic on oral and maxillofacial trauma revealed that the mode of change in dynamics varies by country. Two main factors contribute to this variation. The first factor is the differences in regional characteristics and lifestyles, or so-called national characteristics. Regional factors may include whether the study area is urban or local, differences in medical system for oral and maxillofacial trauma, convenience of transportation, and access to medical care. National characteristics such as alcohol consumption rates, daily exercise habits, and dietary patterns may also be contributory. The second factor involves policy differences such as lockdowns and emergency declarations. Another possible factor is to what extent a lockdown or emergency declaration restricts a citizen’s behavior, and its impact varies depending on the laws of each country. To draw more generalized conclusions on these dynamic changes during the pandemic, additional reports from more countries are essential.

Shimane University Hospital located in Shimane Prefecture, Japan, the region of interest for this study, is a level 1 trauma center that includes oral and maxillofacial trauma. Among the super-aged societies in Japan, Shimane Prefecture is a regional city with a particularly aged population^18^. Therefore, Shimane University Hospital is the cornerstone of trauma care, covering an area with a population of approximately 700,000. In addition, an emergency declaration was issued on April 16, 2020 for all regions in Japan. Japan’s emergency declaration included a request for residents to refrain from leaving their homes, except when necessary to maintain their livelihoods, and to cooperate as necessary to prevent infection, a system that was not as strongly enforced as lockdowns in other countries. Because of the relaxed restrictions, citizens could receive necessary medical care across prefectural borders. Therefore, this study is important as it examines the state of oral and maxillofacial trauma care during an infectious disease pandemic in a developed country, which is expected to become a super-aged society in the future.

Therefore, the aim of this study was to examine the effect of the COVID-19 state of emergency declaration on the dynamics of patients with oral and maxillofacial trauma in Shimane prefecture, a hyper-aged society in a mountainous area of Japan.

## Methods

### Ethical declarations

This study was approved by the Medical Research Ethics Committee, Shimane University Faculty of Medicine (approval number: 20151201–1). Written informed consent was obtained from all participants prior to their participation in the study. Also, all methods were performed in accordance with Declarations of Helsinki.

### Data collection

This study included patients who visited the Department of Oral and Maxillofacial Surgery and Maxillofacial Trauma Center, Shimane University Hospital, between April 1, 2012 and April 30, 2023. Eligible subjects were those seeking treatment for oral and maxillofacial trauma; those with missing data were excluded. The study employed a retrospective observational design, and patient information was obtained from electronic medical records.

### Background data

Data collected from the electronic medical records included the following: age (years), sex (male/female).

### Types of oral and maxillofacial trauma

Data on trauma were obtained according to the following classification: injury status (bruise, laceration [mucosa/skin], traumatic temporomandibular joint arthritis, tooth injury, maxillofacial fracture [alveolus, maxilla, mandible, nasal bone, naso-orbito-ethmoidal fracture, orbital wall, zygoma and zygomatic arch, multiple maxillofacial fractures]).

### Treatment of oral and maxillofacial trauma

Treatment of oral and maxillofacial trauma was categorized into the following treatment methods: bruise, laceration, and traumatic temporomandibular joint arthritis (wound treatment or follow up); tooth injury (wound treatment, crown restoration, extraction, temporary sprint, temporary fixation or follow up); maxillofacial fracture (extraction, temporary sprint, temporary fixation, closed reduction, extraoral fixation, intermaxillary fixation, open reduction and rigid fixation, wound treatment or follow up).

### Referral origin

The classification of the referral source is dental/medical clinic, direct visit, Emergency and Critical Care Center/Advanced Trauma Center, and other departments.

### Mode of transportation to the hospital

The mode of transportation to the hospital was divided into the following categories: ambulance, doctor helicopter, and on foot.

### Cause of injury

The cause of injury was divided into the following categories: fall, slip down, sports, traffic accident, violence, work-related accidents, and others.

### Statistical analysis

For patient background factors, continuous variables were checked for normality using the Shapiro–Wilk test. Descriptive statistics were expressed as the mean (standard deviation) and number of cases (%). Group comparisons were performed between two groups, before and after the COVID-19 pandemic, and t-tests or chi-square tests were performed depending on the type of variable. Interrupted time series analysis was employed to analyze the impact of the COVID-19 pandemic on patient dynamics in patients with oral and maxillofacial trauma^19^. Generally, interrupted time series analysis is the preferred analysis method as a robust quasi-experimental approach in the absence of a control group. Interrupted time series studies have shown that split regression analysis is effective in estimating intervention effects. In this study, the dependent variable Y was the item with significant differences in between-group comparisons, and the model for the number of occurrences per month was constructed as follows:

1 $$E\left( {{\text{Yij}}} \right) \, = \upbeta_{0} + \, \upbeta_{{1}} {\text{time}}_{{{\text{ij}}}} + \, \upbeta_{{2}} {\text{Z}}_{{{\text{ij}}}} + \, \upbeta_{{3}} {\text{Z}}_{{{\text{ij}}}} {\text{time}}_{{{\text{ij}}}}$$

where Y represents the number of cases, and time denotes the number of cases per month from April 2012 to April 2023. Z is a variable set to 0 before the COVID-19 pandemic (until April 15, 2019) and to 1 after the COVID-19 pandemic (after April 16, 2019), and the last variable is the product of Z and time. Since we aimed to reflecting the change in trend (change in slope) due to the COVID-19 pandemic, we considered the shift in patient dynamics when Z_ij_Time_ij_ had a significant probability of *P* < 0.05. One of the greatest strengths of interrupted timeseries studies is their intuitive graphical presentation of results. The visual inspection of the series over time is the first step in analyzing time series data. Such an analysis enables the examination of whether the time trend changed in dynamics before and after an intervention (in this study, a novel coronavirus pandemic). Additionally, Poisson regression analysis was used to create the model since the data were counts of rare events. Statistical analysis was performed using SPSS (version 27; SPSS IBM Corp., Armonk, NY, USA). Two-tailed *P*-values were calculated for all analyses, with an alpha level of significance set at 0.05.

## Results

### Patient characteristics

The data was collected from 1203 patients, including 770 (64.0%) males and 433 (36.0%) females, with a mean age of 39.5 years (standard deviation, 30.7). The most frequent traumatic conditions were tooth injury (n = 229, 19.0%), followed by mucosa laceration (n = 219, 18.2%) and zygoma and zygomatic arch fractures (n = 125, 10.4%). Among treatment methods, open reduction and rigid fixation was performed in 337 (28.0%) patients, and 332 (27.6%) patients received wound treatment. Referrals from an Emergency and Critical Care Center/Advanced Trauma Center accounted for 928 (77.1%) patients, and 826 (68.7%) patients were referred on foot as the method of hospital visit. Slip down was the leading cause of injury, with 593 (49.3%) cases. The patient background characteristics are presented in Table 1.

**Table 1:** Patient characteristics for all data (n = 1203).

Item	Category		n (%) or mean [standard deviation]	
Age			39.5 [30.7]	
Sex	Male		770 (64.0)	
	Female		433 (36.0)	
Traumatic condition	Bruise		51 (4.2)	
	Laceration	Mucosa	219 (18.2)	
		Skin	107 (8.9)	
	Traumatic TMJ^1^ arthritis		20 (1.7)	
	Tooth injury		229 (19.0)	
	Maxillofacial fracture	Alveolus	47 (3.9)	
		Maxilla	37 (3.1)	
		Mandible	94 (7.8)	
		Nasal bone	108 (9.0)	
		NOE^2^	16 (1.3)	
		Orbital wall(s)	94 (7.8)	
		Zygoma and zygomatic arch	125 (10.4)	
		Multiple maxillofacial fractures	56 (4.7)	
Treatment mode (n = 1203)	Wound treatment	Bruise, laceration and Traumatic TMJ^1^ arthritis	299 (24.9)	332 (27.6)
		Tooth injury	24 (2.0)	
		Maxillofacial fracture	9 (0.7)	
	Follow up	Bruise, laceration and Traumatic TMJ^1^ arthritis	95 (7.9)	227 (18.9)
		Tooth injury	50 (4.2)	
		Maxillofacial fracture	82 (6.8)	
	Crown restoration			51 (4.2)
	Extraction	Tooth injury	32 (2.7)	39 (3.3)
		Maxillofacial fracture	7 (0.6)	
	Temporary sprint	Bruise, laceration and Traumatic TMJ^1^ arthritis	1 (0.1)	12 (1.0)
		Tooth injury	7 (0.6)	
		Maxillofacial fracture	4 (0.3)	
	Temporary fixation	Tooth injury	66 (5.5)	99 (8.2)
		Maxillofacial fracture	33 (2.7)	
	Closed reduction (including Nasal bone reduction)			99 (8.2)
	Extraoral fixation			1 (0.1)
	IMF^3^			6 (0.5)
	OR-IF^4^			337 (28.0)
Referral origin	Dental/medical clinic		130 (10.8)	
	Direct visit		62 (5.2)	
	Emergency and Critical care Center/Advanced Trauma center		928 (77.1)	
	Other departments		82 (6.8)	
Method of visit a hospital	Ambulance		344 (28.6)	
	Doctor helicopter		32 (2.7)	
	On foot		826 (68.7)	
Cause of injury	Fall		129 (10.7)	
	Slip down		593 (49.3)	
	Sports		178 (14.8)	
	Traffic accident		188 (15.6)	
	Violence		37 (3.1)	
	Work related accident		26 (2.2)	
	Others		52 (4.3)	

^1^temporomandibular joint, ^2^naso-orbital-ethmoid, ^3^intermaxillary fixation, ^4^open reduction internal fixation.

### Comparison between groups before and after the pandemic

Significant differences were observed in age. Regarding trauma status, significant differences were observed among laceration cases, including nasal bone, orbital wall, zygomatic bone, and zygomatic arch fracture cases. A significant difference was observed in close reduction (including nasal bone reduction) in treating fracture cases, as well as in tooth extraction and wound care among non-fracture patients. Regarding the referral origin, a significant difference was observed in dental/medical clinic and Emergency and Critical Care Center/Advanced Trauma Centers. The mode of transportation to the hospital showed significant differences in ambulance and doctor helicopter usage. Trauma status displayed a significant difference among laceration cases. The cause of injury was significantly different among sports, traffic accidents, and work-related accidents. Details of the group comparisons are presented in Table 2.

**Table 2:** Group comparison of each item before and after the pandemic (n = 1203).

Item	Category		n (%) or mean [standard deviation]		*P*-value
			Before pandemic (n = 831)	After pandemic (n = 372)	
Age			38.1 [30.1]	42.7 [31.9]	0.02*^a^
Sex	Male		537 (64.6)	233 (62.6%)	0.52^b^
	Female		294 (35.4)	139 (37.4%)	
Traumatic condition	Bruise		35 (4.2)	16 (4.3)	1.00^b^
	Laceration	Mucosa	169 (20.3)	50 (13.4)	0.01*^b^
		Skin	77 (9.3)	30 (8.1)	0.58^b^
	Traumatic TMJ^1^ arthritis		17 (2.0)	3 (0.8)	0.15^b^
	Tooth injury		156 (18.8)	73 (19.6)	0.75^b^
	Fracture	Alveolus	35 (4.2)	12 (3.2)	0.52^b^
		Maxilla	29 (3.5)	8 (2.2)	0.28^b^
		Mandible	65 (7.8)	29 (7.8)	1.00^b^
		Nasal bone	65 (7.8)	43 (11.6)	0.04^b^*
		NOE^2^	13 (1.6)	3 (0.8)	0.42^b^
		Orbital wall(s)	56 (6.7)	38 (10.2)	0.05*^b^
		Zygoma and zygomatic arch	74 (8.9)	51 (13.7)	0.01*^b^
		Multiple maxillofacial fractures	40 (4.8)	16 (4.3)	0.77^b^
Treatment mode	Wound Treatment		256 (30.1)	76 (20.4)	0.0001^b^
	Follow up		150 (18.1)	77 (20.7)	0.27
	Crown restoration		39 (4.7)	12 (3.2)	0.24
	Extraction		21 (0.3)	18 (4.8)	0.03^b^
	Temporary sprint		12 (1.4)	0 (0)	0.019^b^
	Temporary fixation		68 (8.2)	31 (8.3)	0.93
	Close reduction (including nasal bone reduction)		55 (6.6)	43 (11.5)	0.003^b^
	Extraoral fixation		1 (0.1)	0 (0)	0.5
	IMF^3^		6 (0.7)	0 (0)	0.1
	OR-IF^4^		222 (26.7)	115 (30.9)	0.13
Referral origin	Dental/medical clinic		74 (8.9)	56 (15.1)	0.002*^b^
	Direct visit		44 (5.3)	18 (4.8)	0.78^b^
	Emergency and Critical Care Center/Advanced Trauma Center		661 (79.5)	267 (71.8)	0.004*^b^
	Other departments		51 (6.1)	31 (8.3)	0.17^b^
Method of visit a hospital	Ambulance		221 (26.6)	123 (33.1)	0.02*^b^
	Doctor helicopter		28 (3.4)	4 (1.1)	0.02*^b^
	On foot		581 (69.9)	245 (65.9)	0.18^b^
Cause of injury	Fall		92 (11.1)	37 (9.9)	0.62^b^
	Slip down		397 (47.8)	196 (52.7)	0.12^b^
	Sports		142 (17.1)	36 (9.7)	0.001*^b^
	Traffic accident		116 (4.0)	72 (19.4)	0.02*^b^
	Violence		27 (3.2)	10 (2.7)	0.72^b^
	Work related accident		23 (2.8)	3 (0.8)	0.03*^b^
	Others		34 (4.1)	18 (4.8)	0.54^b^

^a^t-test, ^b^chi-square test, *P < 0.05.

^1^temporomandibular joint, ^2^naso-orbital-ethmoid, ^3^intermaxillary fixation, ^4^open reduction internal fixation.

### Changes in patient dynamics before and after the pandemic based on interrupted time series analysis

**Figure 1:**
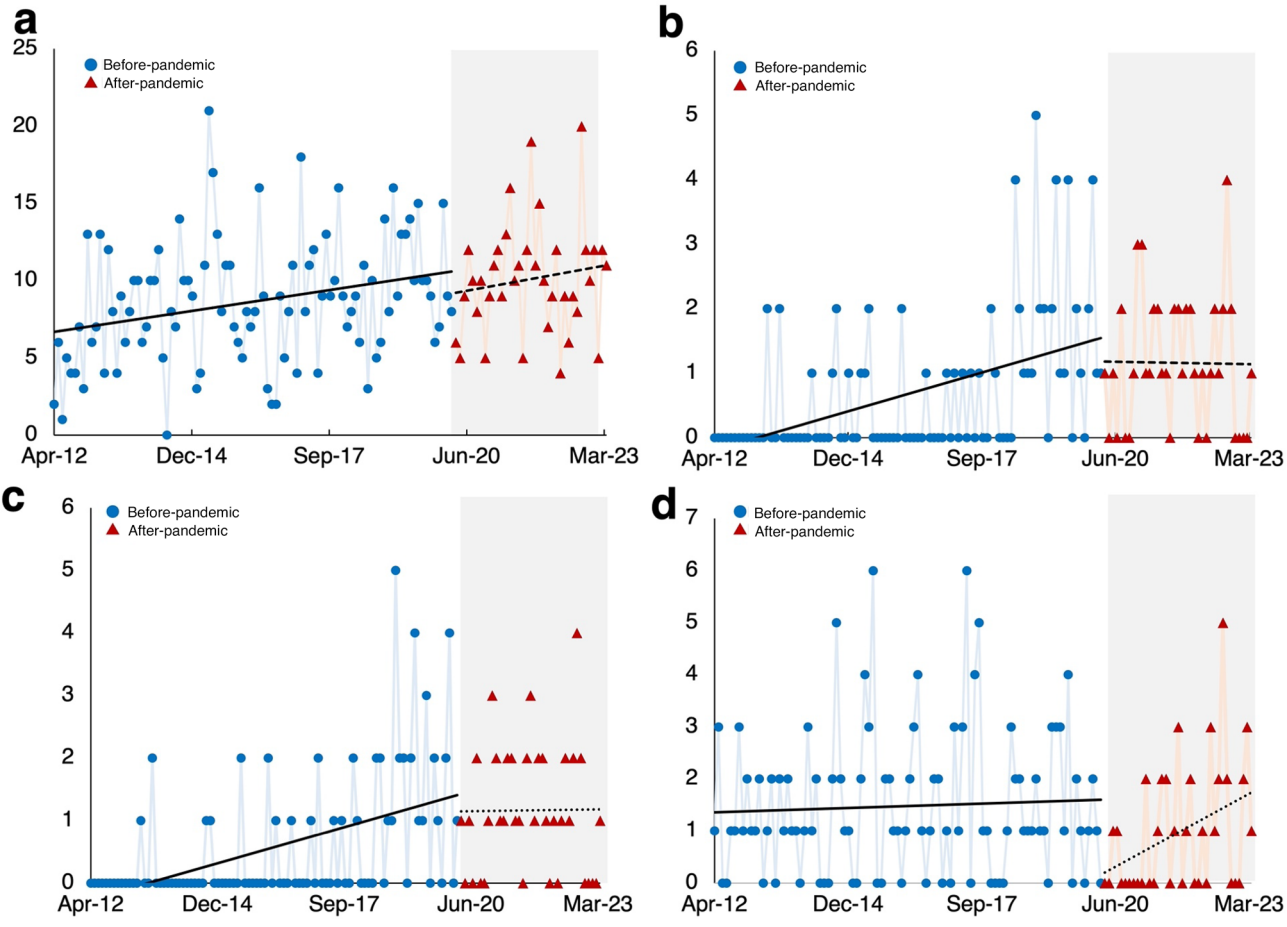
Number of cases over time by stratification. (**a**) Total number of traumatic cases; (**b**) nasal cases; (**c**) number of closed reductions (including nasal bone reduction) in fracture patients; and (**d**) number of sports-related injuries.

The split time series analysis revealed no significant change in the overall number of patients; however, significant dynamic changes were found in nasal bone fracture cases, close reduction (including nasal bone reduction) for maxillofacial fracture patients, and sports as the cause of injury (Fig. 1 and Table 3).

**Table 3:** Changes in patient dynamics before and after the pandemic based on interrupted time series analysis.

		Estimated value	95% confidence interval	*P*-value
*Total traumatic cases (n = 1203)*				
β_0_	Intercept	6.79	5.87–7.85	< 0.01*
β_1_	Time	1.01	1.00–1.01	< 0.01*
β_2_	Z	0.85	0.28–2.57	0.77
β_3_	Z*time	1.00	0.99–1.01	0.98
*Stratified by nasal fracture (n = 1203)*				
β_0_	Intercept	0.11	0.50–0.24	< 0.01*
β_1_	Time	1.03	1.02–1.04	< 0.01*
β_2_	Z	11.99	0.43–332.13	0.14
β_3_	Z*time	0.97	0.94–1.00	0.04*
*Stratified by close reduction (including nasal bone reduction) in maxillofacial fracture patients (n = 577)*				
β_0_	Intercept	0.06	0.02–0.15	< 0.01*
β_1_	Time	1.04	1.02–1.05	< 0.01*
β_2_	Z	17.30	0.60–501.42	0.10
β_3_	Z*time	0.97	0.94–1.00	0.02*
*Stratified by sports (n = 1203)*				
β_0_	Intercept	1.36	0.97–1.91	0.07
β_1_	Time	1.00	1.00–1.01	0.58
β_2_	Z	0.003	0.0001–0.17	0.01*
β_3_	Z*time	1.05	1.01–1.08	0.01*

β: coefficient, Z: a dummy variable indicating the before (coded 0) or after period (coded 1), *P < 0.05.

## Discussion

This study was conducted in a level 1 trauma center in a super-aged community, as previously mentioned, and the data were collected during an “emergency declaration,” a Japanese governmental policy that did not require a mandatory lockdown, which should be considered, given that regional characteristics have a particular impact on this study.

The major finding of this study is divided into three parts. First, the COVID-19 pandemic altered the trend in the number of cases of nasal bone fractures. Nasal bone fractures can result from various causes, including traffic accidents, falls, sports, and violence. A study in Turkey reported a decrease in the number of nasal bone fracture cases during the COVID-19 pandemic compared with the previous year^20^. This aligns with the simple comparison performed in our study, which also indicated an increased percentage during the pandemic, but a decline in dynamics over time. In contrast to the Turkish study, which noted an increase in assault as the cause of nasal bone fractures, our study did not show a similar trend. A meta-analysis on the association between violence and stay-at-home/confinement orders found an increase in incidents of violence, including domestic violence, in response to stay-at-home/confinement orders. Similar results were reported in multiple studies worldwide, making an increase in violence as a cause of nasal bone fractures more plausible^21^. This discrepancy may stem from differences in the extent of the stay-at-home/confinement orders or lockdowns in Japan. Unlike many countries that implemented enforceable lockdowns at the beginning of the COVID-19 pandemic to control the spread of the disease, the Japanese government issued a non-enforceable declaration of a state of emergency. While the declaration of a state of emergency in Japan is believed to have effectively prevented people from going out to high-risk destinations during the pandemic, prolonged declaration of a state of emergency led people to return to their pre-pandemic lifestyle^22^. This dynamic change may be due to sports trauma, but the behavioral restrictions associated with the pandemic may also have contributed to increased frailty and locomotive syndrome in the elderly, leading to an increase in falls^23^. Thus, the declaration of emergency may not have been as coercive as a lockdown, potentially explaining the absence of an increase in violence-related nasal bone fractures.

Second, closed reduction (including nasal bone reduction) mirrored the dynamics of nasal fractures, with an increase in percentage but undergoing a change in trend as a temporal dynamic. A report from the United States reported that during the COVID-19 pandemic, the clinical characteristics of patients with facial fractures remained unchanged, but the use of mandibulomaxillary fixation (MMF), a conservative treatment for mandibular fractures, decreased significantly^24^. However, the cause of the MMF decrease is considered to differ from the change in the dynamics of closed reduction (including nasal bone reduction) in Japan. The authors suggest that the decrease in MMF usage was due to the risk of being unable to perform emergency intubation if COVID-19 disease developed after MMF^25^. In India, all oral and maxillofacial trauma cases were reportedly treated exclusively with closed reduction (including nasal bone reduction) for 6 months after the lockdown^26^. However, at our hospital, the study facility, no situation occurred where the treatment method was changed to closed reduction (including nasal bone reduction) due to the emergency declaration or COVID-19 pandemic. No nosocomial infection occurred among patients or medical staff since the usual treatment method was selected as the first choice. This is inferred as a factor that differs from both India and other countries. Although fully explain the mechanism underlying the results of this study is challenging due to the limited number of previous reports, it is at least possible that the weakly binding restrictions imposed by the declaration of a state of emergency, which were weaker than those of a lockdown, may influence the implemented treatment methods. Simultaneously, these less restrictive laws and regulations may influence the prioritization of medical decisions. Meanwhile, it was suggested that a weakly binding statute might allow for a flexible response that prioritizes medical judgment^27^. The third point, regarding sports as a cause of oral and maxillofacial trauma, suggests that this phenomenon may be unique to Japan. An article on lockdowns and sports trauma in children noted that sports trauma cases were almost completely absent during the lockdown period^28^. A significant decrease in sports-related trauma during a lockdown was also reported in adults^29^. While many papers attribute the reduction in human contact during a lockdown as a factor, Japan showed an increasing dynamic after the declaration of the state of emergency, despite a decrease in percentage. This was attributed to the emergency declaration, in addition to the request of minimizing opportunities for human contact, by avoiding closed, enclosed, and crowded places. This encouraged the elderly to stay indoors, whereas children increased their outdoor activities to reduce the infection risk^30^.

To summarize the three major findings of this study, behavioral restrictions in society may change the lifestyle of individuals, thereby altering the profile of oral and maxillofacial trauma. This result is consistent with that of other reports related to oral and maxillofacial injuries and traumas^31,32^. On the other hand, the fact that the dynamics of the number of oral and maxillofacial trauma cases, including both minor and severe, advanced trauma cases, did not change indicates that oral and maxillofacial surgeons need to maintain the regular healthcare system, even in the event of an acute infectious disease pandemic, because patient needs in oral and maxillofacial trauma care might not change.

A minor finding of this study is that overall oral and maxillofacial trauma cases in Japan were unaffected by the emergency declaration and did not exhibit dynamic changes. Although a strong legally binding lockdown may reduce the risk of emergency room visits, notably in oral and maxillofacial trauma, injuries may increase due to other factors such as increased domestic violence^33^. Therefore, it was deemed that oral and maxillofacial trauma care provided under a declared state of emergency during a pandemic would be better served by maintaining the same medical system and treatment plans as those under normal circumstances.

This study has two limitations. First, it used 10 years of data in the split time series analysis, which assumes that medical standards and systems remained similar during this period. Although in reality, changes may have occurred that were not observed in the data. Second, the region providing data for this study is characterized as a super-aged society in a mountainous area in Japan, potentially limiting the generalizability of the results to other regions. Hence, future multicenter studies are recommended to verify whether similar findings can be obtained in a broader area.

## Conclusion

During infectious disease pandemics, minor trauma cases in the oral and maxillofacial region decrease and trauma cases from outdoor activities increase; however, the overall trend in the number of cases remains stable. During an international pandemic, if a state of emergency is declared without a lockdown, oral and maxillofacial trauma dynamics remain unchanged, underlining the need for regular medical care. The findings of this Japanese study could guide policies for addressing such trauma cases in an aging society during a future pandemic.

## Data Availability

The datasets used and/or analyzed during the current study available from the corresponding author on reasonable request.
